# A Nasal Spray Combining Camostat with a Natural Polysaccharide for the Prevention of Viral Infection via Nasal Mucosal Barrier Formation and Entry Inhibition

**DOI:** 10.3390/ijms27021053

**Published:** 2026-01-21

**Authors:** Yujeong Na, Byeongyong Kim, Dongjin Lee, Jongseo Choi, Sangeun Cho, Kyungmin Lee, Gwanyoung Kim, Eunyoung Cho, Jonggeun Kim, Seong Kug Eo, Sokho Kim

**Affiliations:** 1 Daewoong Pharmaceutical Co., Ltd., 72 Dugye-ro, Pogok-eup, Cheoin-gu, Yongin-si 17028, Gyeonggi-do, Republic of Korea; 2210494@daewoong.co.kr (Y.N.); 2210565@daewoong.co.kr (B.K.); leedj@daewoong.co.kr (D.L.); 2210302@daewoong.co.kr (J.C.); sangeuncho@daewoong.co.kr (S.C.); 1471km@daewoong.co.kr (K.L.); pharmrich@daewoong.co.kr (G.K.); 2 CGBIO Co., Ltd., 52 Sagimakgol-ro, Jungwon-gu, Seongnam-si 13215, Gyeonggi-do, Republic of Korea; jnh4538@cgbio.co.kr (E.C.); jgkim@cgbio.co.kr (J.K.); 3 College of Veterinary Medicine, Biosafety Research Institute and Core Facility Center for Zoonosis Research, Jeonbuk National University, 79 Gobong-ro, Iksan 54596, Jeollabuk-do, Republic of Korea; vetvirus@jbnu.ac.kr; 4 Department of Biohealth, Kunsan National University, 558 Daehak-ro, Gunsan 54150, Jeollabuk-do, Republic of Korea

**Keywords:** prevention, influenza, xanthan gum, camostat, nasal spray

## Abstract

In recent years, numerous researchers have investigated various preventive strategies against respiratory viruses that pose a threat to human health. This study aims to develop a nasal spray formulation based on the natural polysaccharide xanthan gum (XG) and camostat, and to evaluate its dual protective mechanism at the nasal mucosa, the primary entry point for respiratory viral infections. The efficacy of the formulation was assessed through physicochemical characterization, cell-based assays, and animal experiments. Initially, muco-adhesiveness was evaluated by monitoring the drying dispersion area of the test formulation over time on a Petri dish. The combination of XG and camostat exhibited a dispersion area more than ten times larger than that of each component used alone. The antiviral efficacy was demonstrated in both human nasal epithelial cells (HNEc) and an influenza-infected mouse model. The cell-based experiment demonstrated a significant inhibition of viral penetration and replication. Furthermore, suppression of transmembrane protease, serine 2 (TMPRSS2) expression, a key factor in influenza virus entry, was observed in mouse lung tissues. These findings suggest that the Camostat–Polysaccharide Dual-Action Nasal Spray (CPNS), currently under development, holds promise as a non-invasive, first-line barrier to prevent the initial infection and replication of respiratory viruses.

## 1. Introduction

Respiratory diseases have long been considered a major threat to human health and public safety. A key contributor to this threat is the high infectivity of respiratory viruses, which are primarily transmitted via aerosols and can infect the entire respiratory tract once inhaled [1,2]. As is widely recognized, the emergence of the highly infectious and lethal respiratory virus SARS-CoV-2 resulted in a global pandemic with devastating consequences [3]. Another respiratory virus, influenza, is capable of causing both pandemics and seasonal endemics, infecting approximately 10% of adults and up to 30% of young children annually [4,5]. Currently, numerous studies are underway to prevent or treat infections caused by such viruses. The predominant strategies include vaccination and antiviral therapy with small-molecule drugs [6,7]. While these strategies are generally effective, they are often rendered inadequate due to the high mutation rate of viruses [8,9]. Mutant strains can evade immunity conferred by vaccines, and antiviral drugs can lose efficacy due to the development of viral resistance [10,11]. As a result, the importance of personal hygiene and preventive strategies that are less influenced by environmental or societal factors has become increasingly apparent.

As previously mentioned, respiratory viruses are transmitted via aerosols and initiate replication after entering the respiratory tract. In addition, fomite transmission represents an important route of respiratory virus infection and typically occurs via the mucosal surfaces of the upper respiratory tract, including the nasal cavity. If viral entry through the nasal cavity can be effectively controlled, it may be possible to block early-stage infection. To this end, we have been actively investigating novel preventive approaches [12]. Strategies that suppress viral replication or inhibit secondary infection at the early stages may significantly reduce the incidence of respiratory diseases. Although preventive measures such as mask-wearing and surface disinfection are already in place, the addition of pharmaceutical-grade interventions may further enhance disease control. In line with this, we have previously screened potential agents capable of preventing or suppressing viral infection at the nasal mucosa [12]. Based on prior research, we identified the combination of xanthan gum (XG), a natural polysaccharide, and camostat mesylate (hereafter, camostat), a synthetic serine protease inhibitor, as a promising candidate formulation. This led to the development of the current study.

XG is a high-molecular-weight anionic heteropolysaccharide produced by *Xanthomonas campestris* through carbohydrate fermentation. It exhibits shear-thinning behavior, characterized by high viscosity at rest that rapidly decreases under shear stress [13]. XG maintains its viscosity across a wide pH range (about 1–13) and high temperatures (>90 °C), making it suitable for use in food and pharmaceutical applications. In this study, XG was used to enhance the muco-adhesiveness of the test formulation for nasal delivery. Recent other study of XG has demonstrated the utility of XG in artificial mucus flow models, suggesting its potential for forming a mechanical barrier on the nasal mucosa [14].

Camostat developed by Ono Pharmaceutical in Japan, is an oral drug approved for the treatment of pancreatitis and gastroesophageal reflux disease [15]. Camostat inhibits trypsin as well as transmembrane protease serine 2 (TMPRSS2), a key protease involved in viral entry of respiratory viruses such as influenza and SARS-CoV-2 [16]. Accordingly, camostat has recently gained attention as a potential therapeutic agent for COVID-19.

In this study, we aimed to develop a novel intranasal formulation combining the muco-adhesive and mechanical barrier properties of XG with the antiviral potential of camostat, which inhibits TMPRSS2-mediated cellular entry of respiratory viruses such as influenza and SARS-CoV-2. We designate this formulation as Camostat–Polysaccharide Dual-Action Nasal Spray (CPNS). To investigate the dual antiviral actions of CPNS, we conducted a series of physicochemical, cellular, and animal experiments. In the physicochemical study, the muco-adhesiveness of CPNS was evaluated by measuring the dispersion area of dried formulation on a Petri dish over time, and the retention of the formulation in the mouse nasal cavity was also analyzed. In the cellular study, CPNS was applied to human nasal epithelial cells (HNEc) prior to influenza virus infection to assess its antiviral effect, and cytotoxicity was evaluated using murine fibroblasts (L-929). In the animal study, CPNS was administered intranasally prior to influenza virus challenge, and clinical symptoms and TMPRSS2 protein expression levels were subsequently analyzed. The muco-adhesive and mechanical barrier properties of CPNS, together with its ability to suppress TMPRSS2 expression and viral infectivity in both cell-based and animal models, demonstrate the positive efficacy of the test formulation. This study proposes a novel antiviral strategy leveraging the dual mechanism of CPNS to inhibit the onset and progression of respiratory viral infections.

## 2. Results

### 2.1. CPNS Exhibits Muco-Adhesiveness and Retention in the Nasal Cavity

**Figure 1:**
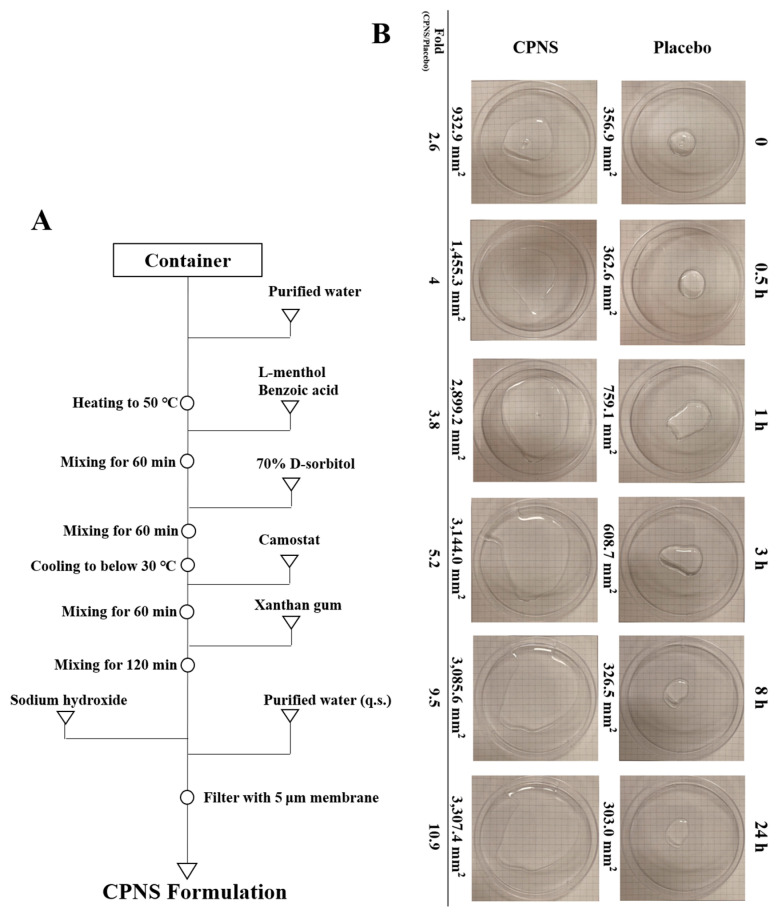
(**A**) Flow diagram of CPNS preparation and (**B**) temporal evaluation of the dispersion area of Placebo and CPNS. Fold change was calculated by dividing the dispersion area of the CPNS-treated sample by that of the Placebo-treated sample.

To evaluate the affinity of CPNS for the nasal mucosa, a drying dispersion area assay was first performed (Figure 1B). Immediately after application, CPNS formed droplets with approximately 2.6 times greater surface area than those of placebo due to its lower surface tension. At 0.5 h post-application, the dispersion area of CPNS was about four times larger than that of placebo, indicating a favorable balance between the formulation’s surface tension, viscosity, and elasticity in response to applied vibrational energy. Over time, the dispersion area of placebo gradually decreased due to water evaporation, whereas CPNS maintained a wide dispersion area despite its initial spread, showing approximately an 11-fold difference in area at 24 h.

**Figure 2:**
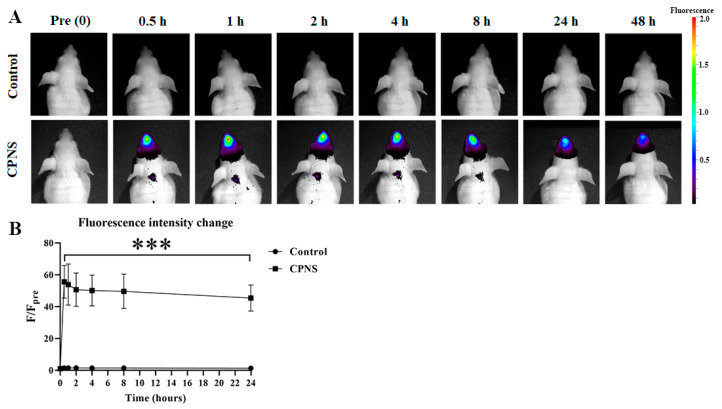
Evaluation of residual fluorescence of CPNS in the nasal cavity. (**A**) Fluorescence images of the nasal region were acquired at 0.5, 1, 2, 4, 8, 24, and 48 h post-administration using the IVIS imaging system. Fluorescence intensity was quantified by selecting a fixed-size ROI within the nasal area and expressed as [p/sec/cm^2^/sr]/[μW/cm^2^]. (**B**) In the control and CPNS-treated group, fluorescence intensities up to 24 h were normalized to the pre-administration (0 h) value. Significance levels are indicated as follows: *** *p* < 0.001. Each time point was compared with the corresponding control group (only fluorescent dye solution-treated group). Data are presented as means ± standard deviation (s.d.); n = 5 per group.

Next, to assess the in vivo film-forming ability of CPNS in the nasal cavity, fluorescently labeled CPNS was administered intranasally in mice and compared with a control group (Figure 2). In the control group, which received only a non-viscous fluorescent dye solution, fluorescence in the nasal cavity rapidly diminished and was undetectable throughout the observation period. In contrast, the CPNS-treated group retained over 80% of the initial fluorescence intensity up to 8 h and showed more than a 50% signal retention even after 24 h. These results indicate that the physical properties of CPNS are advantageous for mucosal adhesion and distribution in the nasal cavity, and for forming a protective barrier over the mucosal surface.

### 2.2. CPNS Is Non-Cytotoxic and Inhibits Viral Infection in Cells

**Figure 3:**
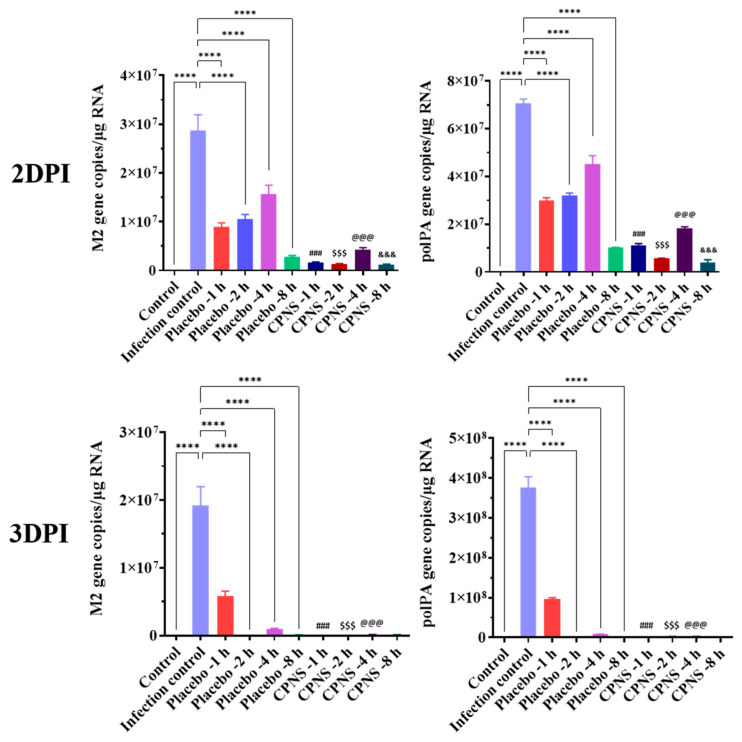
Quantitative evaluation of viral gene expression in influenza virus–infected HNEc using an ALI model at 2 and 3 days post-infection (DPI). All groups except the control group were infected with influenza virus, and the infection control group received no treatment. Whereas the other experimental groups were pretreated with placebo or CPNS at 1, 2, 4, or 8 h prior to viral infection. Significance levels are indicated as follows: **** *p* < 0.0001 compared to infection control, ^###^ *p* < 0.001 compared to placebo -1 h, ^$$$^ *p* < 0.001 compared to placebo -2 h, ^@@@^ *p* < 0.001 compared to placebo -4 h, ^&&&^ *p* < 0.001 compared to placebo -8 h. Data are presented as means ± s.d.; n = 4 per group.

The cytotoxicity of CPNS was assessed according to ISO 10993-5 guidelines. Cell viability was measured following treatment with CPNS at concentrations of 3.13%, 6.25%, 12.5%, 25%, 50%, and 100%, resulting in viability rates of 91.5%, 87.8%, 85.6%, 83.4%, 78.1%, and 71.3%, respectively. The negative control group exhibited 99.3% viability, while the positive control group (ZDEC extract) showed viability rates of 81.4%, 5.6%, 0.7%, and 0.8% at concentrations of 12.5%, 25%, 50%, and 100%, respectively. Based on the ISO 10993-5 threshold, which defines cytotoxicity as cell viability below 70%, CPNS was considered non-cytotoxic at all tested concentrations [17]. To evaluate antiviral efficacy, ALI model using HNEc was employed, simulating the respiratory mucosal environment (Figure 3). The expression levels of the core influenza viral genes M2 and polPA were quantitatively analyzed at 2 and 3 days after viral infection, and the highest expression levels were observed in the untreated infection control group. In contrast, all placebo-pretreated infected groups showed significant reductions in M2 and polPA expression at both time points compared with the untreated infection control group. When CPNS-pretreated groups at each time point were individually compared with placebo-pretreated groups administered under the same timing conditions, significant suppression of M2 and polPA expression was observed in most comparisons. However, at 3 days post-infection, no significant difference was observed between the group pretreated with CPNS 8 h prior to infection and the corresponding placebo-pretreated group. This finding is likely attributable to the muco-adhesive properties of the placebo, which exerted a physical virus-inhibitory effect when administered 8 h before infection, leading to substantial viral suppression by 3 DPI and thereby eliminating differences between the two groups. These ALI model-based results suggest that CPNS has the potential to suppress viral infection more rapidly and robustly than placebo.

### 2.3. Pre-Administration of CPNS Alleviates Pathological Symptoms Caused by Influenza Virus Infection

**Figure 4:**
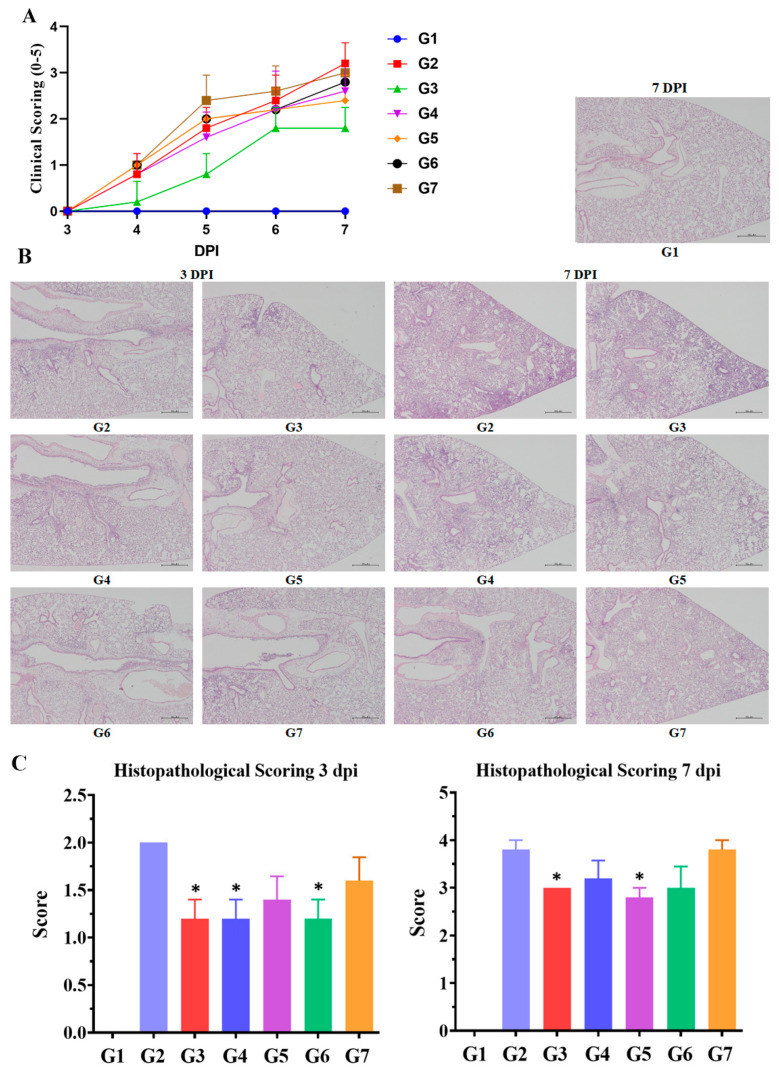
Clinical scoring and histological analysis. G1: Untreated, uninfected control; G2: Untreated infection control; G3: Oseltamivir-treated infection group; G4: CPNS-treated group, 1 h prior to infection; G5: CPNS-treated group, 2 h prior to infection; G6: CPNS-treated group, 4 h prior to infection; G7: CPNS-treated group, 8 h prior to infection. (**A**) Clinical scores recorded from 3 to 7 DPI following influenza virus infection. (**B**) Representative histological images of lung tissues collected after influenza virus infection. The scale bar indicates a length of 500 µm. (**C**) Inflammation scoring graph based on analysis of histological images. Significance levels are indicated as follows: * *p* < 0.05 compared to G2 (untreated infection control). Data are presented as means ± s.d.; n = 5 per group, except for G1, in which four mice were analyzed.

To evaluate the prophylactic efficacy of CPNS against influenza virus infection, an animal infection model was established in which CPNS was intranasally administered to mice at 1, 2, 4, and 8 h prior to viral challenge, and clinical outcomes were monitored for 7 DPI (Figure 4). As shown in Figure 4A, clinical scoring was recorded daily, revealing that the positive control group (G3) exhibited the mildest symptoms overall. Except for the G7 group, all CPNS-treated groups showed clinical scores that were similar to or milder than those of the untreated infection control group (G2) throughout the experimental period. At 7 DPI, the reduction in clinical scores compared to G2 was 44% for G3, 19% for G4, 25% for G5, 13% for G6, and 6% for G7, indicating that CPNS treatment reduced clinical severity by up to 25%. Given that influenza virus-induced pulmonary inflammation is a key pathological outcome, histological analyses of the lungs were conducted at both 3 and 7 DPI (Figure 4B,C). The results demonstrated that, with the exception of G7, all CPNS-treated groups showed reduced pulmonary inflammation compared to G2. Statistically significant reductions in lung inflammation were observed in G4 and G6 at 3 DPI and in G5 at 7 DPI. These findings suggest that intranasal administration of CPNS prior to infection can mitigate both clinical symptoms and virus-induced pulmonary pathology.

### 2.4. Pre-Administration of CPNS Inhibits Viral Replication of Influenza Virus

**Figure 5:**
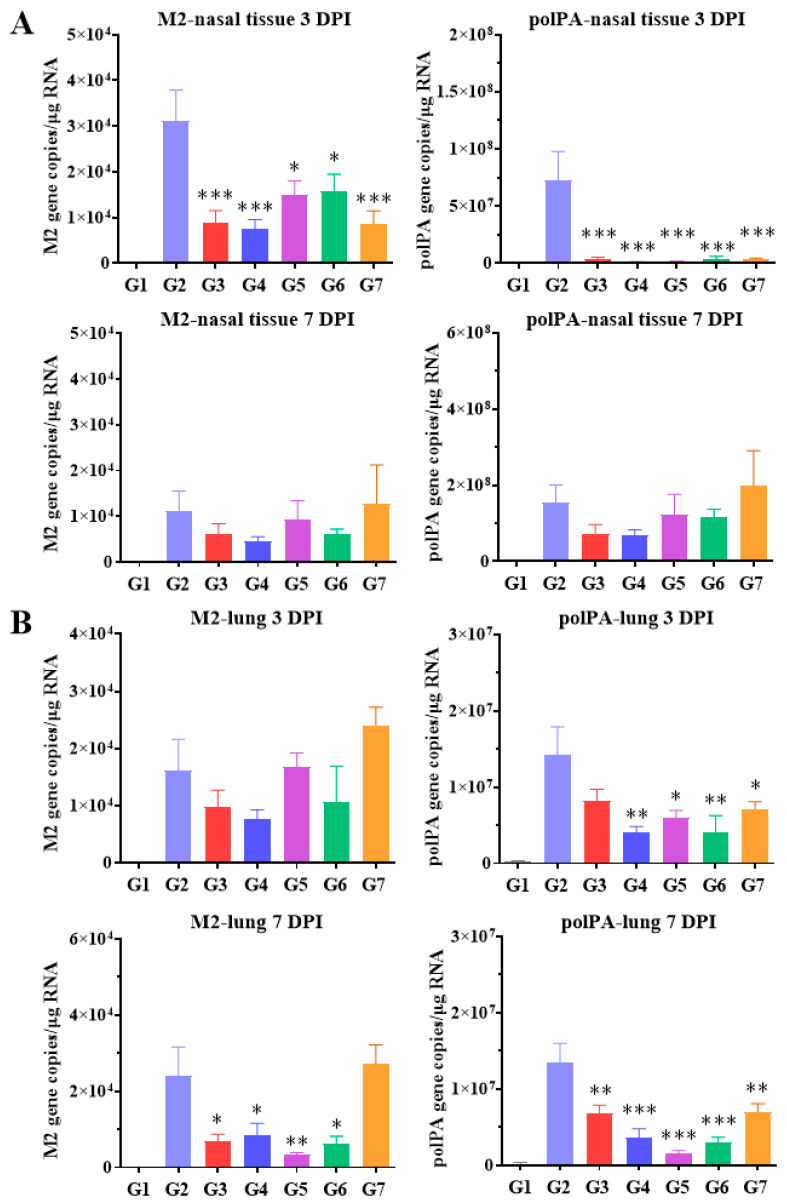
Quantification of viral gene expression in nasal tissue and lung. G1: Untreated, uninfected control; G2: Untreated infection control; G3: Oseltamivir-treated infection group; G4: CPNS-treated group, 1 h prior to infection; G5: CPNS-treated group, 2 h prior to infection; G6: CPNS-treated group, 4 h prior to infection; G7: CPNS-treated group, 8 h prior to infection. (**A**) Expression levels of M2 and polPA genes in nasal tissues at 3 and 7 DPI. (**B**) Expression levels of M2 and polPA genes in lung tissues at 3 and 7 DPI. Significance levels are indicated as follows: *** *p* < 0.001, ** *p* < 0.01, * *p* < 0.05 compared to G2 (untreated infection control). Data are presented as means ± s.d.; n = 5 per group, except for G1, in which four mice were analyzed.

The gene expression levels of influenza virus in the nasal tissue and lungs were analyzed (Figure 5). At 3 DPI, the acute phase of infection, expression levels of the influenza virus genes M2 and polPA in the nasal tissues were significantly reduced in all CPNS-treated groups (G4 to G7) as well as in the oseltamivir-treated group (G3), compared to the untreated infection control group (G2). At 7 DPI, a recovery phase, although the expression levels of M2 and polPA in the nasal tissues appeared to decrease in groups G3 to G6 compared to G2, the differences were not statistically significant (Figure 5A). In lung tissues at 3 DPI, M2 expression levels in groups G3 and G4 were lower than in G2, though not significantly. However, the polPA expression levels in groups G4, G5, G6, and G7 were significantly reduced compared to G2. At 7 DPI, M2 expression in the lungs was significantly decreased in all groups from G3 to G6 compared to G2. The most prominent reduction in polPA expression in the lungs at 7 DPI was observed in G5, and significant reductions were confirmed in all groups from G3 to G7 compared to G2 (Figure 5B). Taken together, significant changes in viral gene expression were observed in CPNS-treated groups compared with G2 at distinct anatomical sites and time points: specifically, in the nasal tissues at 3 DPI and in the lung tissues at 7 DPI. These findings indicate that CPNS effectively acts during the early stage of infection (3 DPI) in the nasal tissues, which represent the upper respiratory tract, thereby suppressing initial viral establishment. In contrast, in the lung tissues representing the lower respiratory tract, CPNS treatment was associated with reduced viral persistence at the later stage of infection (7 DPI), suggesting improved recovery and a favorable disease outcome.

### 2.5. Pre-Administration of CPNS Reduces TMPRSS2 Expression Prior to Influenza Virus Infection

**Figure 6:**
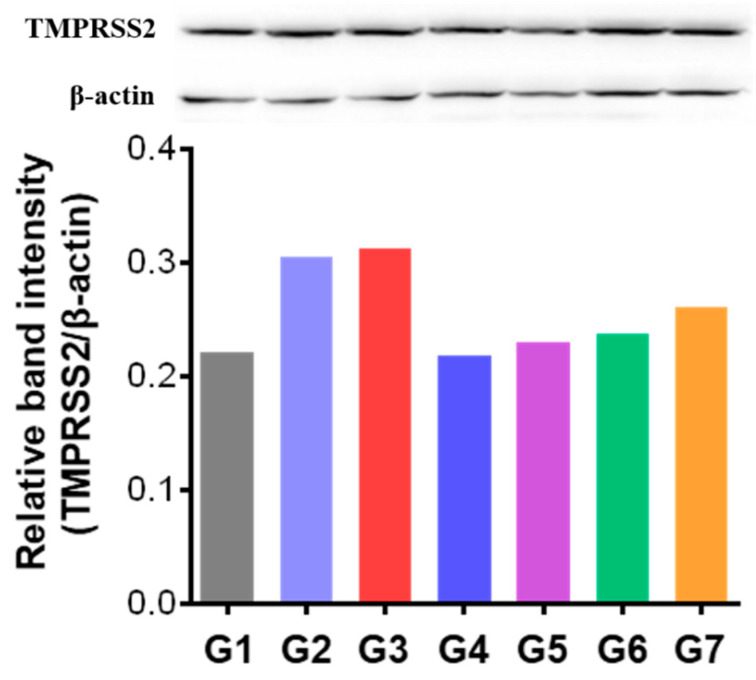
Western blot analysis of TMPRSS2 expression in nasal tissues. G1: Untreated, uninfected control; G2: Untreated infection control; G3: Oseltamivir-treated infection group; G4: CPNS-treated group, 1 h prior to infection; G5: CPNS-treated group, 2 h prior to infection; G6: CPNS-treated group, 4 h prior to infection; G7: CPNS-treated group, 8 h prior to infection. All nasal tissue samples were prepared as group-wise composite samples. Band intensities were quantified using ImageJ software, version 7.12.

To investigate the mechanism underlying CPNS-mediated suppression of influenza virus infection, TMPRSS2 protein expression levels were analyzed. Nasal tissues collected at 7 DPI were pooled by group and used to prepare samples for analysis. Western blotting was performed to evaluate TMPRSS2 expression, and the results were normalized to β-actin expression from the same samples (Figure 6). The findings showed that TMPRSS2 expression levels in all CPNS-treated groups (G4 to G7) were reduced compared to both the untreated infection control group (G2) and the oseltamivir-treated group (G3). Among these, G4 exhibited the greatest reduction, with a 29% decrease in TMPRSS2 expression relative to G2, reaching levels comparable to the uninfected group (G1). These results indicate that the closer the CPNS treatment is administered to the time of infection, the more effectively it suppresses TMPRSS2 expression.

## 3. Discussion

Respiratory viruses primarily invade the human body through the nasal mucosa, and this route of infection plays a critical role in the early replication and transmission of the virus. Viruses such as influenza initiate infection by interacting with TMPRSS2 expressed on mucosal epithelial cells [4], while others like SARS-CoV-2 exploit the angiotensin converting enzyme 2 receptor for cellular entry [18]. Ongoing genetic mutations in these viruses have led to the emergence of new variants with enhanced transmissibility and immune evasion capabilities. Consequently, immunity acquired through vaccination or natural infection alone is often insufficient for complete protection. As a result, adherence to personal protective measures including mask wearing, hand hygiene, and avoidance of close contact has become an increasingly essential strategy for preventing the spread of respiratory viruses. Accordingly, recent studies have increasingly focused on the nasal cavity the primary entry site for respiratory viruses as a critical target for preventing infection [6]. In line with this research trend, the aim of the present study is to evaluate whether our compound, when directly applied to the nasal mucosa, can inhibit influenza virus infection.

Based on our prior in vitro findings that demonstrated the antiviral activity of camostat against influenza, we conceptualized the CPNS system [12]. Camostat exerts its antiviral effects via molecular biological mechanisms as a small molecule drug and is established as an oral formulation. However, it may not exert sufficient local effects on the nasal mucosa. We focused on the muco-adhesive and moisturizing properties of the naturally derived polysaccharide xanthan gum (XG), as well as its ability to form a protective barrier—features that could physically reduce viral infection in the nasal cavity. Leveraging these material characteristics, we developed CPNS as a formulation that combines physical barrier formation and molecular interference against viral entry mechanisms for mucosal application.

To first assess the physical barrier function of CPNS, we evaluated its spread ability (Figure 1B). Compared to XG, CPNS demonstrated broader dispersion and sustained coverage due to enhanced adhesion. This effect is likely attributed to the amphiphilic nature of camostat’s molecular structure—its hydrophilic guanidine and ester groups, along with hydrophobic benzene rings, reduce surface tension [19,20]. Considering the harsh environment of the nasal cavity due to mucociliary clearance and airflow, a formulation like CPNS, which spreads widely and adheres well due to low surface tension, is considered advantageous. This result aligns with our in vivo imaging analysis using fluorescently labeled CPNS, which showed prolonged retention in the nasal cavity (Figure 2). The physical barrier function of CPNS is consistent with the description in the original CPNS patent [21].

When CPNS was applied to an ALI model designed to mimic the respiratory system, significant reductions in M2 and polPA gene expression were observed at 2 and 3 days after viral infection compared to G2 (Figure 3). The placebo formulation, containing XG as the major component but lacking camostat, also reduced M2 and polPA expression in the ALI model, consistent with our previous findings [12]. When CPNS-pretreated groups at each time point were individually compared with placebo-pretreated groups administered under the same timing conditions, significant suppression of M2 and polPA expression was observed in most comparisons. The observed decrease in viral gene expression induced by CPNS indicates both physical and molecular inhibitory effects, which may reduce the probability of infection and associated symptoms. As noted in many studies, the nasal cavity serves as the primary entry route for respiratory viruses, leading to the development of numerous intranasally administered therapeutics [22,23]. Reducing the viral load that penetrates the nasal cavity could lower the risk of severe infection. Accordingly, a growing number of intranasal prophylactic products are being developed [24], and CPNS appears to possess potential preventive capabilities against respiratory virus infections. In subsequent in vivo experiments, CPNS-treated mice exhibited milder clinical symptoms and reduced pulmonary inflammation upon viral challenge (Figure 4). Furthermore, M2 and polPA gene expression in nasal and lung tissues was generally reduced compared to the untreated control group (Figure 5). Comparing viral gene expression between 3 DPI and 7 DPI, we found that CPNS significantly suppressed nasal viral gene expression at 3 DPI (early or acute phase of infection), while it suppressed lung viral gene expression more strongly at 7 DPI (later or recovery phase). This suggests that CPNS exerts its intended early-phase defense at the nasal mucosa. Additionally, CPNS-induced downregulation of TMPRSS2, a critical host protein involved in viral entry, was observed in nasal tissues (Figure 6). The reduction in TMPRSS2 induced by CPNS was confirmed at the molecular level, indicating that CPNS is capable of inhibiting viral infection not only through physical suppression of viral entry mediated by its physicochemical properties, but also through molecular-level interference with viral infection pathways. Collectively, these results indicate that CPNS forms a non-invasive, prophylactic first-line barrier capable of blocking initial viral infection and subsequent replication.

CPNS has passed cytotoxicity testing and shows potential for expansion to other respiratory viruses beyond influenza. It may also be applicable to other mucosal infections and various anatomical sites, supporting the possibility of broader clinical utility. Protective immunity induced by prior infection, vaccination, or hybrid immunity relies heavily on immune responses in the upper respiratory tract to defend against common respiratory viruses such as influenza and SARS-CoV-2. Therefore, CPNS, which acts at the level of the upper respiratory tract, may have broad applicability.

## 4. Materials and Methods

### 4.1. Preparation of CPNS Formulation

The CPNS formulation used in this study was prepared as follows: Initially, 0.2 mg/mL of L-menthol and 0.6 mg/mL of benzoic acid were added to purified water and mixed while maintaining the temperature at 50 °C. Separately, a 70% D-sorbitol solution was prepared at a concentration of 35 mg/mL and was also maintained at 50 °C during mixing. Once the two solutions were fully mixed, the combined solution was cooled to 25 °C. Subsequently, camostat was added at a final concentration of 0.35 mg/mL along with 0.087 mg/mL of sodium hydroxide to adjust the solution to pH 4. Afterward, XG was dissolved into the solution at a concentration of 0.25 mg/mL. Purified water was then added to adjust the final volume to 1 L. The resulting mixture was stirred thoroughly and filtered through a 5 μm membrane filter to complete the preparation of CPNS. Figure 1A presents a flow diagram summarizing the CPNS formulation procedure described above in a simplified and accessible manner. The preparation of CPNS was carried out with reference to our existing patent [21]. All materials used in this CPNS formulation were provided by research center of Daewoong Pharmaceutical (Yongin, Republic of Korea).

### 4.2. Viruses

Flu influenza virus strain FluV/A/PR8/34/H1N1 was used for all in vivo and in vitro experiments. All viruses were propagated via inoculation into the allantoic sac of 9-day-old embryonated chicken eggs using standard procedures [25]. After passage in 9–10-day-old embryonated eggs, the allantoic fluid was harvested and stored at −70 °C. The viral titer was measured 48 h post-inoculation and calculated as plaque-forming units per milliliter (PFU/mL) according to the method described in previous study [26]. For in vivo studies, the virus diluted to a concentration of 1 lethal dose 50 (LD50; total 30 μL) was administered intranasally to mice through the left nasal cavity. The 1 LD50 was determined to be 150 PFU/mL.

### 4.3. Mouse Preparation and Handling

10 seven-week-old female Balb/c nude mice were purchased fom orient bio (Seongnam, Republic of Korea) for intranasal film formation experiment. 64 six-week-old female C57BL/6 mice were purchased from Samtaco, Inc. (Osan-si, Republic of Korea) for influenza infection experiment. The mice were housed in filter-top microisolator cages and maintained under controlled conditions: 23 °C, 55% humidity, and a 12 h light/dark cycle. They were provided with water and feed ad libitum. During the first seven days, we monitored the mice for health status, including feeding behavior and growth. All animal experiments were approved by the Institutional Animal Care and Use Committee (IACUC) of Asan institute for life Sciences and HLB bioStep and conducted in compliance with Institutional Biosafety Committee guidelines and the ARRIVE guidelines (Approval code: Asan Institute for Life Sciences IACUC 2025-40-094, HLB bioStep IACUC 24-HB-0840).

### 4.4. Physicochemical Study

#### 4.4.1. Drying Dispersion Area Assay

To evaluate muco-adhesiveness, 1 mL of CPNS or the placebo (CPNS formulation without camostat) was dispensed onto a 100 mm-diameter Petri dish using a micropipette. The sample was then evenly distributed by applying horizontal vibration at 10 Hz using a Voltexer-2 Genie (Scientific industries, Bohemia, NY, USA). The plates were incubated at 37 °C to allow drying, and the dispersion pattern was assessed by measuring the spread area of the remaining liquid over time. Images were taken at 0, 0.5, 1, 3, 8, and 24 h after application [27], and the surface area of the spread liquid was quantified using ImageJ software, version 7.12 (NIH, Bethesda, MD, USA).

#### 4.4.2. Intranasal Film Formation and Retention Assay in Balb/c Nude Mice

Balb/c nude mice were anesthetized using inhaled isoflurane (Piramal critical care, Bethlehem, PA, USA). Before administration, background images were captured using an in vivo imaging system, IVIS (PerkinElmer, Waltham, MA, USA). CPNS was fluorescently labeled by mixing with Cyanine 5.5 amine (Lumiprobe, Cockeysville, MD, USA) in distilled water (D.W.) at a volume ratio of 10:1. The mixture was incubated at room temperature overnight to ensure complete conjugation. After completion of the fluorescent dye conjugation reaction, the reaction mixture was transferred to an Amicon^®^ ultrafiltration tube and centrifuged at 3000× *g* for 15 min at 4 °C. The filtrate containing unconjugated free fluorescent dye was discarded, and the retained fluorescently conjugated fraction was collected. The retained fraction was then transferred to a dialysis membrane and dialyzed against distilled water at 4 °C with buffer exchange performed every 1 h for a total of 6 h to ensure complete removal of residual unconjugated dye. Only the fluorescently labeled CPNS was used as the test formulation in subsequent experiments. Measurement of the fluorescence content in the final conjugated test material confirmed that 0.4% of the fluorescent dye was bound. For the control group, a fluorescent dye solution was prepared without CPNS by dissolving Cyanine 5.5 amine in D.W. to achieve the same final fluorescent dye concentration of 0.4% at room temperature. A fluorescently labeled CPNS formulation and a fluorescent dye solution which served as control were intranasally administered at a dose of 20 μL per mouse (0.25 mg/kg, left nostril only) in groups of five mice per treatment. After administration, fluorescence images were acquired at 0.5, 1, 2, 4, 8, 24, and 48 h using the IVIS. Fluorescence intensity in the nasal region was quantified by selecting a region of interest (ROI) of fixed dimensions [28].

### 4.5. Cellular Study

#### 4.5.1. Cytotoxicity Assay

The cytotoxicity test was conducted in accordance with ISO 10993-5 (Biological Evaluation of Medical Devices, Part 5: Tests for In Vitro Cytotoxicity). Briefly, CPNS was extracted at a ratio of 1 mL of MEM medium (Hyclone, Logan, UT, USA) supplemented with 10% fetal bovine serum (FBS) per 0.2 g of sample. The extraction was performed at 37 °C for 24 h under agitation in a 5% CO_2_ incubator. The resulting extract was serially diluted with the same medium to yield six concentrations (3.13%, 6.25%, 12.5%, 25%, 50%, and 100%) for testing. The negative control was prepared by subjecting the MEM medium alone (without test substance) to the same extraction conditions. The positive control was prepared using ZDEC polyurethane film (Hatano RI, Kanagawa, Japan) according to standard cytotoxicity protocols, with an extraction ratio of 1 mL of MEM per 0.1 g of material. This extract was tested at four concentrations (12.5%, 25%, 50%, and 100%). The cell line used for cytotoxicity evaluation was NCTC Clone 929 (L-929), purchased from ATCC (Manassas, VA, USA). Cells were cultured in monolayers and treated with 100 μL of each test concentration. The plates were incubated at 37 °C for 24 h in a 5% CO_2_ incubator. Following incubation, the medium was removed, and cell viability was assessed using an MTT assay kit (Sigma-Aldrich, St. Louis, MO, USA). Absorbance was measured at 570 nm using a microplate reader (Agilent, Santa Clara, CA, USA). A reduction in cell viability to below 70% of the negative control was interpreted as indicating cytotoxicity.

#### 4.5.2. Virus Infection in HNEc Under Air–Liquid Interface (ALI)

HNEc were purchased from PromoCell (Heidelberg, Germany) and cultured at 37 °C in a 5% CO_2_ incubator using the PneumaCult™-Ex Plus Medium (STEMCELL technologies, Vancouver, BC, Canada). For seeding, 1 mL of PneumaCult™-Ex Plus Medium was added to the basal chamber of each well in a 12-well plate, and cells were seeded onto the apical chamber at a density of 1 × 10^5^ cells/cm^2^. The Transwell^®^ insert plates (Corning, NY, USA) were incubated at 37 °C until HNEc reached confluence (approximately 2–3 days) [29]. To initiate ALI conditions, the apical medium was removed from the Transwell insert, and the basal medium was replaced with 1 mL of PneumaCult™-ALI Maintenance Medium (STEMCELL technologies, Vancouver, BC, Canada). The medium in the basal chamber was replaced every 48 h, and the cells were maintained for 21 days prior to treatment. For the viral inhibition assay, 140 μL of placebo (CPNS formulation without camostat) or CPNS was applied to the apical side of the ALI-cultured HNEc at 1, 2, 4, or 8 h prior to influenza viral infection (0.001 MOI). Total RNA was extracted 48 and 72 h after infection for the quantification of M2 and polPA gene expression. Briefly, cells harvested from each well were used for total RNA extraction using Wizol™ Reagent (Wizbio-solutions, Seongnam, Republic of Korea). The extracted RNA was subjected to M2 and polPA expression analysis as described in the PCR section.

### 4.6. Animal Study and Histological Analysis in C57BL/6 Mice

CPNS was administered intranasally using a syringe designed for spray dispersion, whereas oseltamivir (Cipla, Mumbai, India) was administered orally after being dissolved in distilled water. CPNS was administered at a dose of 20 μL per mouse (one dose per administration), starting prior to viral infection and continued three times daily at 8 h intervals. Oseltamivir, used as the positive control (100 μg in 20 μL per mouse), was administered beginning 1 h after viral infection and given twice daily at 12 h intervals. Only group G1 consisted of 4 mice, which served as uninfected controls and received no viral infection or treatment. All other groups, including controls and treatment groups, included 10 mice each and were subjected to viral infection. The experimental groups were defined as follows: G2, the negative control group, was infected but received no treatment; G3, the positive control group, received oseltamivir; G4 through G7 received CPNS starting at 1, 2, 4, and 8 h prior to viral infection, respectively. All experiments continued until 7 days post-infection (7 DPI). Clinical scoring was performed daily according to the following criteria: 1, slight ruffling of fur; 2, ruffled fur with reduced mobility; 3, ruffled fur, reduced mobility, and rapid breathing; 4, ruffled fur, reduced mobility, huddled appearance, and rapid and/or labored breathing indicative of pneumonia; 5, death. Animals exhibiting signs of pneumonia or experiencing greater than 20% body weight loss were euthanized. On days 3 and 7 post-infection, five mice per group were euthanized for tissue sampling, and G1 mice were euthanized at 7 DPI. Inhalation anesthesia was used for all mice, followed by exsanguination via transection of the abdominal aorta and inferior vena cava. Nasal tissue and entire lungs were collected; half of each sample was stored at approximately −70 °C for PCR and Western blot analyses, while the remaining tissue for histology was fixed in 4% formalin, dehydrated in graded ethanol, embedded in paraffin, sectioned at 4 μm, and stained with hematoxylin and eosin (H&E; Thermo Scientific/Merck, Waltham, MA, USA). Lung damage was scored using a previously established system [30]: minimal, scattered inflammatory cells in the parenchyma; mild, aggregated inflammatory cells in <1/3 of the parenchyma; moderate, aggregated inflammatory cells in 1/3 to 2/3 of the parenchyma; and severe, aggregated inflammatory cells in >2/3 of the parenchyma.

### 4.7. Western Blots

Nasal tissues collected at 7 DPI were washed three times with phosphate-buffered saline (PBS). Prior to protein extraction, all nasal tissue samples were pooled by group and prepared as composite samples. Total protein was extracted by incubating the samples for 1 h in RIPA buffer (GenDEPOT, Katy, TX, USA) supplemented with phosphatase and protease inhibitor cocktail (GenDEPOT, TX, USA). The lysates were centrifuged at 15,000× *g* for 15 min, and protein concentrations were determined using a bicinchoninic acid (BCA) assay kit. Equal amounts of protein were denatured by heat treatment at 100 °C for 5 min and separated by electrophoresis on a 10% SDS–polyacrylamide gel. Following electrophoresis, proteins were transferred onto polyvinylidene difluoride (PVDF) membranes (Bio-Rad, Hercules, CA, USA), which were then blocked with 5% skimmed milk for 1 h at room temperature. The membranes were subsequently incubated overnight at 4 °C with primary antibodies against TMPRSS2 and β-actin (Abcam, Cambridge, UK), both diluted 1:1000 in 5% skimmed milk. After washing three times with PBS containing 0.1% Tween-20 (PBS-T), the membranes were incubated for 1 h at room temperature with HRP-conjugated secondary antibodies (goat anti-mouse or anti-rabbit IgG, GenDEPOT, Katy, TX, USA) diluted 1:5000 in 5% skimmed milk. Signal detection was performed using the SuperSignal West Dura Extended Duration Substrate (Thermo Scientific, Waltham, MA, USA), and chemiluminescence was visualized using a WSE-6200 Luminograph II imaging system (ATTO, Tokyo, Japan). Quantification of protein band intensities was performed using ImageJ software, version 7.12 (NIH, USA).

### 4.8. Real-Time RT-PCR

Total RNA was extracted individually from lung and nasal tissue samples collected at 3 and 7 DPI, following a previously described protocol [31]. RNA extraction was performed using Wizol™ Reagent (Wizbio-solutions, Seongnam, Republic of Korea). The extracted RNA samples were subjected to quantitative real-time reverse transcription PCR (qRT-PCR) using a CFX96 Real-Time PCR Detection System (Bio-Rad, Berkeley, CA, USA). Complementary DNA (cDNA) was synthesized from total RNA using the High-Capacity cDNA Reverse Transcription Kit (Applied Biosystems, Foster City, CA, USA). Each PCR reaction (20 μL total volume) contained 2 μL of template cDNA, 10 μL of 2× Premix Ex Taq, and 200 nM of each primer and probe. The primer and probe sets were as follows: for the *M2* gene, forward primer 5′-CTT CTA ACC GAG GTC GAA ACG TA-3′, reverse primer 5′-GGT GAC AGG ATT GGT CTT GTC TTT A-3′, and probe [FAM]5′-TCA GGC CCC CTC AAA GCC GAG-3′[BHQ1]; for the *polPA* gene, forward primer 5′-CGG TCC AAA TTC CTG CTG A-3′, reverse primer 5′-CAT TGG GTT CCT TCC ATC CA-3′, and probe [HEX]5′-CCA AGT CAT GAA GGA GAG GGA ATA CCG CT-3′[BHQ1]. PCR cycling conditions consisted of an initial denaturation at 95 °C for 30 s, followed by 45 cycles of 95 °C for 5 s and 60 °C for 20 s. No-template controls were included in every assay. Each sample was analyzed in at least duplicate to ensure reproducibility, and specificity of amplification was confirmed by melting curve analysis. Viral load was quantified by absolute copy number using a standard curve generated from known concentrations of viral cDNA, and the results were expressed as viral RNA copies per microgram of total RNA. Data were analyzed using Bio-Rad CFX Manager version 2.1.

### 4.9. Statistical Analysis

Parametric or non-parametric statistical procedures were used to compare the mean values of the parameters across groups. For parametric comparisons, one-way ANOVA followed by Dunnett’s multiple comparison test was applied. In cases where non-parametric methods were required, the Kruskal–Wallis rank-sum test and Mann–Whitney U test were used. For the experimental results, parametric statistical procedures were used for analyzing viral titers, while non-parametric methods were applied for histopathological analysis. We used GraphPad Prism 6 software to analyze and summarize the results. Statistical significance was determined at *p* < 0.05.

## 5. Conclusions

Blocking viral entry at the nasal stage provides a unique opportunity to prevent infection at its source. Even low levels of virus exposure in daily life can lead to severe disease when immunity is compromised. Therefore, there is a need for nasal formulations capable of preventing viral infection at an early stage. The preclinical results from this study demonstrated the potential of CPNS as an effective inhibitor of influenza virus. As a nasal spray formulation with high muco-adhesiveness and TMPRSS2-suppressing properties, CPNS effectively blocked viral entry and replication at the nasal mucosa. Its dual mechanism of action supports the feasibility of CPNS as a non-invasive, self-administered prophylactic agent suitable for large-scale use during pandemics, particularly to protect high-risk populations from transmission by asymptomatic individuals.

## Data Availability

All data are presented in the manuscript and are available from the authors.

## Abbreviations

The following abbreviations are used in this manuscript:

XG	Xanthan gum
HNEc	human nasal epithelial cells
TMPRSS2	Transmembrane protease, serine 2
CPNS	Camostat–Polysaccharide Dual-Action Nasal Spray
Camostat	Camostat mesylate
PFU	Plaque-forming units
LD50	Lethal dose 50
ROI	Region of interest
ZDEC	Zinc Diethyldithiocarbamate
